# Supplementary material for: Are carbon emissions trading and green financial instruments synergistic? -Comprehensive quantitative research based on content analysis
Source: PLoS One. 2024 Mar 7;19(3):e0298601. doi: 10.1371/journal.pone.0298601 (PMC10919601; doi:10.1371/journal.pone.0298601)
Supplement: S1 Data — (ZIP) [file pone.0298601.s001.zip › Data/地级市Z_F工作报告.zip!/╕╩╦α/╛╞╚¬╩╨/└┤╫╘╣1⁄2╓┌║┼í╛┬φ┐╦╩2╛▌═°í┐_2015.docx]

各位代表：现在，我代表酒泉市人民政府向大会作工作报告，请予审议，并请各位政协委员和其他列席人员提出意见。

　　2014年政府工作回顾

　　2014年，在省委、省政府和市委的坚强领导下，我们认真贯彻党的十八大和十八届三中、四中全会以及习近平总书记系列重要讲话精神，坚持“五做五加强”工作思路，深入实施“3341”项目工程，全面深化各项改革，实现了经济社会持续稳步发展。预计，全市完成生产总值634亿元，增长9%；固定资产投资1003亿元，增长21%；社会消费品零售总额156.7亿元，增长12.5%；财政总收入93.6亿元，增长9.6%，其中公共财政预算收入32.2亿元，增长20.7%；财政总支出117.6亿元，增长5.5%；城镇居民人均可支配收入25083元，农民人均纯收入12283元，分别增长11.9%和13.2%。

　　———做精一产取得实效。设施农业、特色产业快速发展，肃州国家级和敦煌省级现代农业示范区带动效应增强，科技示范园区达到120个，高效节水和规模养殖占比提高，分别达到82%和78%，特色产业占农民收入的62%，农业增加值增长5.3%。

　　———做强二产结构优化。新能源首位产业地位更加突出，六个循环经济百亿元产业链培育壮大，八大战略新兴产业加速形成，九大传统产业集群逐步崛起，工业增加值增长10.2%，首位产业和循环经济分别增长6.8%和10.9%，占工业增加值的比重提高4.9个百分点。

　　———做大三产步伐加快。华夏文明传承创新区建设全面推进，文化产业增加值增长23%；文化旅游加速融合，物流、信息、电商等服务业态快速发展，第三产业对经济增长的贡献率达到35%，比上年提高3个百分点。

　　———做美城市亮点纷呈。国际明星城和戈壁明珠城建设有序推进，敦煌市和肃州区总寨镇、金塔县航天镇列入省级新型城镇化试点。各县市区城区美化、亮化、扩容改造工程建成，人均公共绿地面积达到11.2平方米，城市品位提升，承载能力增强，城镇化率达到55.1%，提高1.5个百分点。

　　———做实民生成效显著。城镇登记失业率控制在3.01%，居民消费价格指数涨幅控制在2.6%以内，城乡低保、五保供养等社保待遇和财政补助标准全面提高，校安工程、保障房建设、安全饮水等省市政府承诺的惠民实事全面完成，各项约束性指标控制在省定目标以内。

　　需要说明的是，面对市场环境变化和经济下行压力，我们在分析研判的基础上，报经市人大常委会批准，对去年年初确定的部分预期目标作了调整。生产总值增幅由12%调整为8.5%，固定资产投资增幅由27%调整为21%，社会消费品零售总额增幅由14%调整为12.5%。这是主动适应经济发展新常态、准确把握改革发展稳定平衡点、统筹协调经济运行质量和着力改善民生做出的普遍性调整。一年来，我们加强对重点产业、重点行业的协调服务，自加压力，负重奋进，着力推动经济发展提质增效和社会事业全面进步，主要做了以下工作：

　　1．全力稳增长，强化项目支撑效应。坚持把项目建设作为稳增长的重要支撑，改进项目投资管理办法，完善重大项目并联审批制度，狠抓项目谋划论证和争取衔接，加快审核报批节奏，实行重大项目督查通报制度，全市开工建设重大项目1974个，其中亿元以上项目307个，投资对经济增长的贡献率达到53%，提高0.5个百分点。全面推进基础设施建设，兰新高铁、瓜敦一级公路建成通车，酒航一级公路奠基，敦格铁路、白明高速加快推进，敦煌机场扩建、酒额铁路前期工作进展顺利。敦煌水资源合理利用与生态保护项目稳步实施，酒泉循环经济产业园等园区水资源引蓄工程开工建设，灌区续建配套、病险水闸加固、河道治理、湿地保护等项目全面完成。强化招商引资责任机制，大力推进产业链招商，新增落地项目124个，在建项目达到829个，引进到位资金911亿元。

　　2．强力促改革，激发增强市场活力。全面落实民间投资和非公经济市场准入激励政策，放宽企业工商注册登记条件，新增各类市场主体1.1万户，私营企业注册资本增长109.3%，非公经济实现增加值315.4亿元，同比增长18%。大力推进投融资体制改革，设立金融创新发展专项基金，开辟小微企业贷款绿色通道，加大支农贴息贷款落实力度，金融信贷增长20%以上。奥凯种机、祁连药业上市进展顺利，敦煌种业增发股票募集资金4.8亿元，金龙矿业、荣泰橡胶等32家企业在甘肃省股权交易中心和上海股权托管交易中心挂牌，直接融资22.3亿元。农村土地承包经营权确权登记颁证试点工作全面推进，新增土地流转面积6.7万亩，累计流转50万亩，占家庭承包经营面积的25%。财政预决算公开、政府采购“采管分离”、国库集中支付、公务卡统一结算等财政改革有序推进。“营改增”试点范围扩大，非税收入管理进一步规范。调整工商、质监、食药、卫生、计生等部门管理体制，市直部门精简6%，县市区工作部门减少13%，酒泉经济技术开发区管理体制初步建立，启动飞天数字影院改制工作，经济社会发展活力进一步增强。

　　3．努力调结构，优化提升产业层次。着眼提高发展质量，推动产业改造升级，三次产业结构调整为13.4：47：39.6。认真落实各项强农惠农富农政策，实施农林水利项目485个，新增日光温室、设施蔬菜、特色林果10万亩，设施农业达到43万亩，高效田占到耕地面积的60%以上；新增肉牛1万头、肉羊100万只，肉羊饲养量达到700万只；新建改建产业化龙头项目13个，农产品加工增值率达到60%以上。制定出台“止滑稳增”十一条措施，着力解决企业资金融通、电力外送和产品运销等瓶颈问题，新增风电装机245万千瓦、光电装机33.8万千瓦、水电装机7万千瓦，电力装机规模突破1100万千瓦，年发电量174.5亿千瓦时，增长2.7%。国家风电设备质检中心建成，省光伏设备质监中心获批，风光电装备制造形成全产业链生产能力。麻黄滩等8个330千伏升压站和输变电工程建成，酒泉原湖南依800千伏特高压直流输电及瓜州常乐电厂即将核准建设。水木紫荆、浩海煤化工、恒亚水泥等一批项目建成投产，循环经济产业链对工业增长的贡献达到55.7%。改扩建大型商贸中心和特色商业街4个，巨龙农产品市场列入全省大型公益性市场，柳沟煤炭物流园搬迁改造具备转场条件。敦煌国家级文化产业示范园区正式获批，丝绸之路（敦煌）国际文化博览会列入国家“一带一路”规划，锁阳城、悬泉置、玉门关申遗成功，敦煌雅丹国家地质公园列入国土资源部2015年世界地质公园推荐名单。举办“玄奘之路”国际商学院戈壁挑战赛等28项节会赛事，开通了“敦煌号”旅游列车和曼谷直达敦煌包机等航线，旅游接待人数、旅游收入分别达到1380万人次和120亿元，均增长26%。投入科技创新引导资金1000万元，实施市级科技项目44个，新建院士专家工作站4个，实现技术合同交易额20.圆亿元，科技进步对经济增长的贡献率达到54%。

　　4．着力强基础，推动城乡一体发展。新城区三期、高铁片区等控制性详规全面完成，市区控制性详规和全市村镇规划实现全覆盖。汉唐文化新区、北大河生态治理完成年度建设任务，新城区二期“一纵五横”道路全部建成，酒泉、玉门、瓜州高铁站及配套工程建成投运。祁雪路等5条道路和北新花园等13个老旧小区改造完成，城区公厕全部免费开放，新增热电联供面积126万平方米、天然气用户2万户，累计达到930万平方米、4.5万户。全国园林城市、国家卫生城市和全国文明城市创建深入推进，新增绿化面积376万平方米，12345市民服务中心和敦煌智慧城市平台投入使用。大力推进美丽乡村建设，新续建楼居式社区化中心村22个，改造农村危旧房5492户，新建改造农村公路657公里，建成村容村貌整治示范点225个。完成人工造林10.缘万亩、封滩育林8万亩，森林覆盖率达到5.缘%。实施减排项目40个、污染治理项目15个，酒泉第二污水处理厂完成主体工程，县市城区污水处理厂全部建成运营，单位工业增加值用水量下降20%，万元GDP能耗下降3.5%。

　　5．竭力惠民生，促进社会和谐进步。坚持把保障和改善民生作为政府工作的出发点和落脚点，全市投入惠民实事和民生资金28.4亿元，占总支出的35.4%。落实扶贫资金3亿元，实施“联村联户”和扶贫攻坚项目600个，输转移民乡村劳动力3.3万人，移民人均纯收入达到4300元，增长25%。增加创业贷款担保基金2600万元，发放创业贷款3.5亿元，扶持创业实体8413个，新增城镇就业2.8万人。企业退休人员基础养老金月均增加员苑猿元，失业金月均提高89元，农村五保供养标准提高510元，新农合、城镇居民医疗保险、城乡低保标准平均提高15%。新建城乡日间照料中心75个，累计建成146个，覆盖48%的城市社区和26%的行政村。启动实施27种重特大疾病医疗救助制度，城乡医疗救助和临时救助措施进一步完善。分配入住保障性住房3459套，新建保障性住房4666套，发放租赁补贴7900户。实施薄弱学校改造工程89个，消除危房11.4万平方米，建成农村寄宿制学校标准化食堂20个，敦煌、阿克塞义务教育均衡发展通过国家评估认定。西北师范大学敦煌学院成立，招收全日制本科生95名。公立医疗机构全面实行药品网上集中采购，市医院精神病区、儿科病区、全科医生临床培养基地和三级医院综合服务能力建设项目完成主体工程，单独两孩生育政策全面落实。酒泉市博物馆建成，酒泉美术馆等公益文化设施向市民开放，新建“图书漂流”服务点68个。玉门、瓜州、敦煌、阿克塞等县市体育馆建成投用，承办国家级体育赛事4项、省级2项，酒泉体育健儿在全国全省体育赛事中取得优异成绩。妥善处置3起人间肺鼠疫、“8.26”重大交通事故、“12.10”供热管网泄漏等突发公共事件。食品药品日常监管加强，安全生产形势总体平稳，四项控制指标全面下降。“两个共同”示范县创建和民族乡村项目对口帮扶深入推进，民族地区经济社会发展步伐加快。加大矛盾纠纷排查调处力度，完善社会治安防控体系，严厉打击各类违法犯罪活动，维护了社会大局和谐稳定。妇女、儿童、老龄、残疾人事业不断发展，气象、地震、地质灾害监测预报服务水平提高，统计、档案、史志、人防、边防、外事、台侨、驻外联络经协等事业取得新进步。各位代表，一年来，我们扎实推进群众路线教育实践活动，着力解决存在的“四风”突出问题，健全用制度管人、靠程序办事的长效机制，强化履职责任考核和政务服务督查，政府工作效能不断提升。全面落实中央“八项规定”、国务院“约法三章”和省市委“双十条”规定，会议、文件分别压缩32.3%和28%，“三公”经费压减32.9%。坚持依法行政，严格执行政府决策程序和议事规则，自觉接受人大法律监督、工作监督和政协民主监督，办理人大代表意见建议和政协提案278件，办结率均达到96%。深化行政审批制度改革，清理废止规范性文件68件、重新修订63件，取消合并下放行政审批事项46项，保留的行政审批和行政许可项目纳入电子政务监察系统，全部进驻行政服务中心公开办理。认真贯彻落实党风廉政建设主体责任，持续开展建设工程招投标、土地和矿产资源招拍挂、政府采购、财政资金使用等领域专项检查，加强对预算执行、民生社保等专项资金和政府投资项目的行政监察、审计监督，健全公共资源交易流程，实现了100%进场交易，公共资源交易中心率先在全省通过ISO9001管理体系认证。

　　各位代表！成绩的取得，是市委正确领导，市人大、政协监督支持，全市人民奋力拼搏的结果。在此，我代表市人民政府，向全市各族人民，向奋战在各条战线的人大代表、政协委员和广大干部群众，向各民主党派、工商联、人民团体、离退休老同志，向驻酒解放军指战员、武警官兵和公安干警，向中央、省属驻酒单位以及所有关心、支持酒泉发展的各界人士表示衷心的感谢和崇高的敬意！在肯定成绩的同时，我们清醒地认识到，全市经济社会发展还存在一些突出矛盾和问题。一是经济持续健康运行的基础不稳固，农业产业化水平低，工业资源开发占比高，现代服务业发展基础薄弱，稳增长、调结构、促转型的任务艰巨。二是招商引进项目资金到位率偏低，项目建设与资源配置的协同性不够，制约项目落地的因素还比较多。三是支柱产业税收贡献度下降，地方财政收入质量不高，财政保障和偿债压力较大。四是城市基础设施功能薄弱，公共服务设施建设滞后，与城镇化的快速发展不相适应。五是大宗农产品价格波动较大，农民持续增收面临较大压力。六是一些干部解决问题的思路不宽、手段不多，工作作风还需进一步转变。这些问题，既是制约我市当前经济社会发展的难点，也是我们做好今后工作的重点，我们将进一步健全机制，强化措施，认真加以解决。

　　2015年工作总体部署

　　2015年，是全面贯彻落实党的十八届四中全会精神的开局之年，也是“十二五”规划的收官之年和“十三五”规划的编制之年。面对世界经济复杂多变、国内经济下行压力持续加大的形势，我们要紧紧抓住党中央、国务院“丝绸之路经济带”战略的启动实施和定向调控微刺激政策逐步释放的机遇，加快酒泉新能源基地和敦煌国际文化旅游名城建设，争取更多的政策支持和项目支持，促进经济社会各项事业均衡发展。今年政府工作的总体要求是：以党的十八大、十八届三中、四中全会和习总书记系列重要讲话精神为指导，深入贯彻省第十二次党代会和市委三届六次、七次全委会精神，牢牢把握稳中求进、改革创新、依法行政工作总基调，坚持以提高经济发展质量和效益为中心，主动适应经济发展新常态，按照“五做五加强”的总体思路和“一手抓发展、一手抓廉政”的工作部署，在稳定增长中优化结构，在深化改革中创新发展，在扩大内需中推进开放，在改善民生中促进和谐，努力推动经济社会转型跨越发展，勇于挑大梁、做标杆、走前列。

　　全市经济社会发展主要目标是：地区生产总值增长8.5%，固定资产投资增长20%，社会消费品零售总额增长12%，公共财政预算收入增长12%，城镇居民人均可支配收入增长9%，农民人均纯收入增长11%，万元生产总值能耗降低3%，主要污染物排放完成约束性指标。重点抓好七个方面的工作：

　　一、深化改革抓重点、增活力坚持把改革创新贯穿于经济社会发展的各个领域各个环节，进一步增强发展的内生动力，充分发挥市场在资源配置中的决定性作用。大力扶持发展混合所有制经济，组建市级产权交易服务中心，建立健全股权交易市场，开展碳排放交易试点工作；深入推进工商注册便利化，推行电子营业执照和全程电子化登记管理，全面消除各种隐性壁垒，支持民间资本进入基础设施、市政建设、社会发展等领域，力争非公经济占GDP比重提高1.5个百分点。进一步理顺环境保护行政管理体制，建立公共机构节能效果与公共经费安排联动机制，落实生产、生活、生态空间形态用途管制措施，为解决生态环境领域的深层次矛盾和问题提供体制保障。建立规范透明的预算管理制度，全面推进部门预算和“三公”经费公开，清理政府性债务，规范财税优惠政策，推行政府向社会购买公共服务，扩大“营改增”试点范围，努力做到依法理财。以农村房地合确权发证为先导，全面启动不动产统一登记工作，科学界定市区两级管理事权，明确经济技术开发区功能定位，完善功能区规划，抓好经济技术开发区新型工业化示范基地建设，促进经开区由产业主导型向产城融合型发展。

　　二、立足发展抓项目、强后劲坚持把项目作为培育产业企业、积蓄发展后劲的强大引擎，确保实现项目数量和质量“双提升”。准确把握国家支持全局性、基础性、战略性重大项目的投资导向，谋划储备一批产业发展、基础设施、社会事业和生态建设项目，力争更多的项目列入国家和省上“十三五”规划。加快推进额哈铁路、敦格铁路、酒航一级公路、白明高速公路建设，争取柳敦高等级公路、敦煌机场扩建开工，抓好酒额铁路、敦煌高铁、敦当高速、百立通用机场前期工作。加快敦煌水资源合理利用与生态保护项目建设，继续实施封滩育林、禁牧休牧、风沙口治理等生态修复工程，力争《祁连山水源涵养区生态保护和综合治理规划》年内启动。积极适应经济转型升级新要求，围绕主导产业和配套产业链，更加突出引进“高、新、强”项目，完善招商项目落地协调推进机制，优化项目建设环境和服务保障，提高招商项目履约率、开工率和资金到位率。

　　三、做精一产抓规模、提效益继续以服务城市、富裕农民为目标，突出建基地、育龙头、占市场，创建品牌，强化基础，力争农业增加值增长5.3%。深入推进农村各项改革，全面开展土地承包经营权确权登记颁证，积极推进设施农牧业产权确权颁证试点，探索建立农村产权交易平台，引导土地经营权有序流转，大力推进规模经营。深化水权制度建设，开展疏勒河流域水权试点工作，全面落实差别水价政策，着力提高水资源利用效率和效益。认真实施“365”现代农业发展行动计划，加快肃州国家现代农业示范区建设和敦煌省级现代农业示范区升级。持续推进优势特色产业标准化、规模化生产，集中建设一批规模连片生产小区和标准化规模养殖场，特色种植面积增长7%以上，温室大棚和牛羊饲养量均增长8%以上。加快“无公害”、“绿色”、“有机”农产品和“产地地理标志”认定认证工作进度，力争“三品一标”农产品生产面积提高3个百分点，确保农产品质量抽检合格率达到99%以上。强化农业生产资料和农产品市场监管，鼓励支持种业发展和交易市场建设。加强动植物疫病防控工作，确保农牧业和林业生态安全。推进农业产业化经营，扶持壮大农产品精深加工和运销龙头企业，着力打造肃州百亿元农产品加工产业园，农产品加工增值率提高2个百分点。继续打好移民扶贫攻坚战，突出基础设施建设、主导产业培育、发展能力提升三项重点，强化“双联”推动、精准扶贫、多元投入，力争移民人均纯收入增长20%以上，减少贫困人口2万人。

　　四、做强二产抓创新、求实效持续做强壮大新能源首位产业，改造提升传统产业，多元发展新兴产业，力争工业增加值增长8%。启动编制风电三期规划，探索光热发电和氢产业等清洁能源新兴领域发展动态，加快风电700万千瓦和光电280万千瓦前期工作，新增风电装机50万千瓦、光电装机70万千瓦，确保风电二期首批300万千瓦项目并网发电。围绕新能源技术研发、风光电场建设与装备制造互为市场、融合发展，推进装备制造业延链增效，开工新建光伏电池分装胶膜、单晶硅拉棒等项目，新能源及装备制造业占工业的比重达到35%以上。积极开展输配电价改革试点，支持用电大户与发电企业直供直销，力争酒泉—湖南依800千伏直流输变电工程开工建设。认真做好热电联供二期项目前期工作，争取甘电投常乐电厂开工建设，新增水电、生物发电装机10万千瓦。积极争取循环化改造示范项目和高载能企业电价补贴政策，力促万象建材、新哈矿业等项目建成投产，力争广汇煤炭分质利用项目落户酒泉，循环经济占工业的比重达到50%。以新能源、现代服务业等战略性新兴产业为重点，扶持金风科技、巨龙物流港省列骨干企业做大做强，促进敦煌国家信息惠民试点城市建设。积极推进信息化和工业化深度融合，支持传统产业改造提升，鼓励支柱产业集群发展，扶持中小企业扩张规模，引导龙头企业组建产业联盟，深入挖掘小微企业增长潜力，发展规模以上企业40户。积极培育企业创新主体，搭建科技创新平台，加快高新技术的引进吸收和产业化。

　　五、做大三产抓节点、调结构坚持把第三产业作为调整产业结构、培育地方财源的主战场，深入推进服务业综合配套改革，加快文化旅游深度融合，努力打造“丝绸之路经济带”重要节点的商贸物流中心、旅游集散地和旅游目的地，确保第三产业增加值增长9%。大力发展各类户外休闲运动，带动医疗保健、健康养生等高端消费，推动传统服务业升级换档。认真落实金融创新发展奖励政策办法，吸引国内外金融企业和投资基金在酒泉设立独立法人机构，以资本优势带动产业发展、人才聚集。整合集聚物流园区，全面完成柳沟物流园搬迁，加快建设大敦煌、龙德盛等各类商贸专业批发市场。加强海关、商检和口岸建设，争取敦煌航空口岸开放和马鬃山口岸复关，积极推进嘉峪关机场航空口岸开放。抓好巨龙农商网、聚馨飞天网等电子商务平台建设，鼓励共青团组织青年开展网上创业，酒泉电子商务示范区年内投入使用。加快编制酒泉华夏文明传承创新区建设规划和文化产业发展规划，深度挖掘历史文化内涵，全面推进“十三板块”建设和酒泉汉唐文化新区、敦煌国际文化旅游名城建设，扶持发展传媒、演艺、创意、会展、影视、出版等文化业态，力促敦煌旱码头、戈壁绿洲艺术家文化园等文化产业基地早日建成，确保文化产业增加值增长20%以上。积极发展体育产业，办好“玄奘之路”、“非凡足迹”、戈壁马拉松等户外运动。加强与丝绸之路沿线国家和省市区的合作交流，办好第六届中国（甘肃）国际新能源产业博览会、第五届敦煌行·丝绸之路国际旅游节和第一届丝绸之路（敦煌）国际文化博览会等节会。认真组织实施旅游业改革发展意见，编制完成莫高窟—鸣沙山·月牙泉、酒泉卫星发射中心等大景区总体规划，加快欢乐嘉年华西部游乐园、敦煌盛典演艺、丹霞地貌及硅化木地质公园等项目建设，启动阳关、玉门关、金塔沙漠森林公园、瓜州草圣故里等景区晋级建设工作。积极开发文化旅游产品，加大旅游宣传促销，大力开拓客源市场，力争游客接待人数和旅游收入增长20%。

　　六、做美城市抓规划、重管理坚持推进新型城镇化，加快构建以城带乡、城乡一体的新型城乡关系。统筹考虑整体建筑风格、水景绿地体系、民俗文化传承，全面完成酒泉新型城镇化规划、历史文化名城保护规划、经济技术开发区控制性详规编制，完善城市地下管网和地上综合交通等专项规划，着力解决安全隐患和交通拥堵、停车难等问题，推进敦煌市、玉门市“多规合一”国家试点，实现城市、县城、重点小城镇控制性详规全覆盖。加快北大河综合治理、新城区二期道路管网建设，确保北大河河道景观、商业步行街一期等项目全面建成。深入推进全国园林城市、国家卫生城市和全国文明城市创建工作，大力实施庭院绿化、立体绿化和道路绿化，力争城区绿化覆盖率提高1个百分点。加强城市综合执法，巩固提升市容市貌、市场秩序专项整治成果，开展“我爱酒泉”文明礼貌活动，启动“智慧酒泉”建设，完善“数字酒泉”信息系统，提高城管信息化水平。以完善功能、改善环境、凸显特色为重点，强化产业支撑，完善公共服务，落实居住证制度，有序推进农村转移人口市民化，力争城镇化率提高1.5个百分点。加快新农村建设，抓好敦煌市美丽乡村试点县建设，大力开展环境整洁和美丽乡村示范村、示范组创建活动，打造环境优美、生活甜美、社会和美的新农村。

　　七、做实民生抓热点、解难题积极回应人民群众对美好生活的新期待，持续加大民生投入，扎实推进惠民工程，努力提高群众的幸福指数。鼓励支持民族地区加快发展，共同繁荣，共同进步。全面落实就业创业政策，加强技能培训和就业服务，做好高校毕业生、农民工、就业困难人员就业工作，城镇登记失业率控制在4%以内。建立更加公平可持续的社会保障制度，完善城镇职工医疗保险和城镇居民医疗保险政策，做好医疗救助、疾病应急救助制度与其他医疗保障制度的衔接，探索市场化运作养老模式，城乡居民养老保险和医疗保险覆盖面巩固在98豫以上。深化教育领域综合改革，加快改善薄弱学校办学条件，全面消除中小学D级危房，调整优化城乡普惠性学前教育资源配置，支持社会资本兴办学前教育，实现乡镇中心幼儿园全覆盖。扶持发展职业教育，建立职业院校与园区企业有效对接模式。大力推进文化体制改革，完善文化遗产保护与传承工作机制，完成飞天数字影院改制，健全公共文化服务网络。深入实施体育惠民工程，推动文化体育设施免费开放。加快县级医院法人治理结构改革，推进医疗签约服务和医师多点执业，全面推行县乡医疗机构“先诊疗后付费”。扶持发展中医药事业，理顺中医院管理体制，加快中医药产业园建设。认真落实“单独两孩”生育政策，深入开展幸福家庭创建活动，人口自然增长率控制在5.5译以内。强化市场价格监管，确保生活必需品供求基本平衡，居民消费价格涨幅控制在3%以内。积极推进社会治理创新，加强社会稳定风险评估，健全治安防控网络体系，持续开展严打整治和打黑除恶专项斗争，不断提高人民群众安全感。加强法治宣传教育，健全普法责任机制，着力提高全社会的法治意识。建立食品药品原产地追溯制度，强化生产经营全过程监管，确保人民群众食药安全。严格落实安全生产监管责任和企业主体责任，深入开展重点行业领域专项整治，坚决遏制重特大安全事故发生。切实加强环境保护，全面落实减排及污染治理任务，进一步改善环境质量。完善网络舆情监管机制，形成正面引导和依法管理相结合的网络舆论工作格局，凝聚社会正能量。健全应急管理机制，高度重视鼠疫防治，提高应急处置能力。深入开展双拥共建，积极创建全国双拥模范城。继续做好妇女儿童、残疾人、外事、台侨、统计、地震、史志、人防、档案、驻外联络经协等工作。切实解决民生问题，集中力量办好十件实事：一是选拔1000名高校毕业生进企业见习就业；二是实施百所薄弱学校改造工程，新建、维修校舍6万平方米；三是建设“边疆万里数字文化长廊”服务点100个，深入开展“书香酒泉”惠民文化活动；四是建成市医院精神病区、儿科病区、全科医生临床培养基地和三级医院综合服务能力建设项目；五是新建农村公路600公里；六是解决1.2万名农村居民安全饮水问题；七是在酒泉城区新建公益性蔬菜市场3个，在县城和农村集镇新建农贸市场5个；八是新建公共租赁住房2100套、棚户区改造安置房1300套；九是完成城市地下管网普查和规划，科学合理开发建设，切实解决安全隐患，提高供暖、供气入户率；十是继续组织实施“天眼百卡”工程，新建公共部位视频监控1000个，城市道路智能卡口60个，组建公益性岗位治安巡防队15支。

　　各位代表！“十三五”是全面建成小康社会的攻坚阶段，我们将坚持问政于民、问计于民、问需于民，科学论证编制“十三五”规划，研究制定加快经济、社会、文化、生态建设的重大措施，积极谋划对经济发展和结构调整全局带动性强的重大项目，争取更多的政策支持，切实做到科学合理、符合实际，经得起时间检验，鼓舞大家奋斗的信心。

　　全面加强政府自身建设

　　各位代表，全面完成今年的各项目标任务，市政府肩负着重任，承载着厚望。我们将以时不我待的紧迫感、慢进则退的危机感、不负众望的使命感，不断加强自身建设，忠实履行职责，切实做到对人民负责、为人民服务、受人民监督、让人民满意。一是始终坚持解放思想，科学理政，建设学习型政府。立足发展大局，加强形势研判，注重对经济政策、改革措施、发展趋势的学习研究，深化对本地区本行业发展瓶颈、关键环节、发展机遇的准确把握，切实增强抓好经济工作和社会管理的能力，不断提高行政素质和执政水平。加强调查研究，把握和运用好市场经济规律，抓住工作重点，集聚合力，勇于担当，善做善成，提高应对复杂局面的能力。深化市情认识，深入解放思想，不断更新知识层次，转变思维方式，推动管理创新、制度创新、工作创新，确保政府工作更富创造性、更具科学性。二是始终坚持民主决策，依法行政，建设法治型政府。坚持法定职责必须为、法无授权不可为，严格遵守行政组织和行政程序法律制度。坚持科学民主依法决策，完善公众参与、专家论证、风险评估、合法性审查、集体讨论决策程序，建立健全政府法律顾问、决策责任追究及倒查等制度。深化行政执法体制改革，大力推行综合执法，严格实行行政执法人员持证上岗和资格管理制度，健全行政执法和刑事司法衔接机制。认真落实执法全过程记录、重大执法决定法制审核和行政裁量权基准制度，规范行政许可、行政处罚、行政检查等执法行为，扎实稳妥地开展好行政复议试点工作。自觉接受人大、政协监督，加强民主协商建设，广泛听取社会各界意见。坚持以公开为常态、不公开为例外，推进财政预算、公共资源配置、重大建设项目批准实施和社会公益事业建设等领域信息公开。三是始终坚持务实高效，为民执政，建设服务型政府。认真履行政府职能，强化宏观调控、公共服务、市场监管、社会管理、环境保护职责，推动政府职能向创造良好发展环境、提供优质公共服务、维护社会公平正义转变，努力实现好、维护好、发展好人民群众的根本利益。加大简政放权力度，建立市政府部门行政权力清单、责任清单、市级财政专项资金管理清单，深化行政审批制度改革，继续取消和下放行政审批事项，削减前置审批，实行投资项目网上核准，强化并联审批和限时办结制度，努力让审批流程更合理、项目落地更容易、群众办事更便捷。严格执行八项规定和省市各项规定，巩固拓展群众路线教育实践活动成果，健全深入了解民情、充分反映民意、广泛集中民智的工作机制，推动政府工作人员深入基层察民情接地气，切实做到“三严三实”，持续解决“四风”问题。四是始终坚持清廉自律，勤政廉政，建设廉洁型政府。认真执行党风廉政建设责任制，严格落实“一岗双责”、党风廉政建设和从严治党主体责任，深化重点领域、关键环节、重要部位的权力制约和防控，健全用制度管权、管事、管人的长效机制，保证政府权力规范运行。牢固树立过紧日子的思想，严格落实政府性楼堂馆所不得新建、财政供养人员只减不增、三公经费支出只减不增“约法三章”，厉行勤俭节约，反对铺张浪费，严格控制一般性支出，全面推进公车改革，切实把有限的财力用于推动发展和改善民生。强化行政问责、审计监督和效能监察，加大工程建设、土地出让、政府采购、征地拆迁等重点领域的监管力度，实行公共资金、国有资产、国有资源和领导干部履行经济责任情况审计全覆盖，坚决查处行政乱作为、行政不作为、行政慢作为和违纪违法案件。

　　各位代表！实干成就伟业，奋斗铸就辉煌！我们决心在市委的坚强领导下，凝聚全市人民的智慧和力量，锐意进取，真抓实干，攻坚克难，奋力谱写建设富裕文明和谐幸福酒泉的新篇章。
